# Assessing eco-anxiety across the lifespan: A systematic review of current global scales

**DOI:** 10.1016/j.joclim.2025.100595

**Published:** 2025-11-07

**Authors:** Sashka K. Samarawickrama, Sally Richmond, Nerelie C. Freeman, Hannah Kirk

**Affiliations:** a Turner Institute for Brain and Mental Health, School of Psychological Sciences, Monash University, Clayton, Victoria, Australia; b School of Educational Psychology and Counselling, Faculty of Education, Monash University, Clayton, Victoria, Australia

**Keywords:** Eco-anxiety, Climate change anxiety, Assessment, Scales, Systematic review

## Abstract

**Background:**

Eco-anxiety is a growing global concern. While research has sought to understand eco-anxiety and its impact on mental health, there is limited consensus regarding the validity and reliability of eco-anxiety assessment scales. Appropriate scales are needed to enable accurate assessment and effective management of eco-anxiety. The review aims to outline existing eco-anxiety scales across the lifespan, critically evaluate their content, development, psychometric properties, and cross-cultural validity, to identify the most robust scales, and propose recommendations for the future development of eco-anxiety scales.

**Methods:**

PsycINFO, Web of Science, Scopus, PubMed, and Google Scholar were searched (up until May 2025). Articles that developed or validated an eco-anxiety or climate change anxiety scale were eligible. Information regarding the content, development process, psychometric properties, and cross-cultural validation were extracted.

**Results:**

Sixty-seven articles were included in the review. Twelve scales were identified, assessing eco-anxiety (*n* = 4) and climate change anxiety (*n* = 8). Half of the scales were developed for adults (≥18 years) and most were developed in Western/European countries.

**Conclusions:**

The Hogg Eco-Anxiety Scale and the climate anxiety subscale from the Inventory of Climate Emotions were identified as the most robust measures of eco-anxiety and climate change anxiety, respectively. Several limitations of existing eco-anxiety scales were identified, such as insufficient content captured by items, inadequate development processes, minimal reporting of psychometric properties, and poor consideration of participants’ demographic characteristics*.* We propose recommendations to refine the development of future scales to facilitate a more consolidated understanding of eco-anxiety.

## 1 Introduction

The negative impact that human activity has had on the environment is well established, with consequences including climate change, pollution, deforestation, rising sea levels, and loss of biodiversity [1,2]. As a result of the environmental crisis, many domains of humanity are threatened, including health [3]. The impacts of the environmental crisis on physical health, such as heat-related stress and novel infectious diseases are well known [4,5]. In contrast, the impacts of the environmental crisis on mental wellbeing are less clearly understood, though there is increasing recognition by mental health professionals, researchers and policymakers of its importance [6]. For example, in 2022, the United Nations’ Intergovernmental Panel on Climate Change (IPCC) highlighted the mental health consequences of the environmental crisis for the first time, stating with “very high confidence” that climate change endangers mental health and wellbeing, and that the environmental crisis is expected to further threaten mental health [7]. Recent studies acknowledge affective responses, such as anxiety, anger, hopelessness, and sadness, stemming from individuals knowing about the current and future consequences of the environmental crisis [8,9].

Affective responses to the environmental crisis have been termed ‘eco-emotions’ [10,11]. The most commonly reported eco-emotion is eco-anxiety, which describes anxiety about current and future threats to the environment, such as climate change, loss of biodiversity, and pollution [[12], [13], [14], [15]]. Although eco-anxiety is frequently referred to in the media and environmental literature, there is no consensus about definitions or terminology that should be used. Indeed, a range of definitions (e.g., a chronic fear of environmental doom or distress in response to changes in the climate system), overlapping emotions (e.g., eco-grief or eco-anger) and related terminology (e.g., climate change anxiety) are used interchangeably when referring to eco-anxiety [12]. The absence of standardised definitions/terminology have resulted in the use of varied assessment tools for eco-anxiety: ranging from scales that solely evaluate eco-anxiety [8,16] to scales assessing multiple distinct eco-emotions [17]. This variability hinders comparability, complicates the evaluation of the specific impact of eco-anxiety, and introduces risks of tautology [11,12]. Following recent recommendations [12] this review defines eco-anxiety as a ‘chronic fear of environmental doom’, proposed by the American Psychological Association [15]. Eco-anxiety will be the primary term in this review, and climate change anxiety will be included as a subset of eco-anxiety, as recommended in recent work [[12], [13], [14],^18^]. These two terms differ slightly, with eco-anxiety referring to anxiety about an array of environmental problems, such as loss of biodiversity, pollution, and climate change, whereas climate change anxiety refers to anxiety solely about climate change [15,18].

Agreeing upon the definition of eco-anxiety is crucial, but equally important is establishing consensus about standardised scales that should be used to assess eco-anxiety. Without such consensus, quantifying diverse presentations of eco-anxiety across the lifespan and understanding its broader impacts becomes difficult [19]. Additionally, standardised scales may assist in more accurately quantifying the prevalence of eco-anxiety in the community, thus facilitating the identification of vulnerable groups in need of support. Standardised scales would also allow systematic comparisons of findings across studies and enable tracking of eco-anxiety over time. Given the rapid evolution of this field, it is critical to evaluate existing eco-anxiety scales and provide timely recommendations for future scale development, ensuring a unified approach to exploring, understanding and quantifying eco-anxiety.

A small number of reviews have identified that the methodological quality of commonly used eco-anxiety scales is limited; however, these reviews did not evaluate all existing eco-anxiety scales [12,20,21]. One review examined eco-emotion scales in young people [22], but noted that most scales are of low quality, and only 26 % specifically assessed eco-anxiety or climate-change anxiety. As such, their conclusions may not be relevant for scales developed for broader age ranges, nor scales that solely assess eco-anxiety. Collectively, existing reviews provide preliminary evidence that eco-anxiety scales require refinement; however, no review has systematically evaluated the reliability or validity of eco-anxiety scales across the lifespan, nor provided guidance about the most appropriate existing scales for researchers to utilize. Additionally, with the rapid expansion of the field, several new scales and validation studies have not been evaluated by previous reviews.

The present review aims to provide an updated investigation and address gaps in the literature by systematically: (1) mapping and evaluating eco-anxiety scales; (2) examining these scales across the lifespan; and (3) providing recommendations to improve future scales while identifying the most reliable and valid currently available scales.

## 2 Methods

A systematic review was undertaken to address the specified research aims. The review adhered to the Preferred Reporting Items for Systematic Reviews and Meta-Analyses (PRISMA) guidelines [23] and followed the Consensus-Based Standards for the Selection of Health Measurement Instruments (COSMIN) methodology, for systematic reviews of patient-reported outcome measures [24]. The significant methodological differences between studies precluded a meta-analysis.

### 2.1 Search strategy and information sources

The search strategy was informed by previous reviews investigating similar topics [22] but were narrowed down to focus on eco-anxiety and climate change anxiety. To identify relevant studies, the authors searched electronic databases (PsycINFO, Web of Science, Scopus, PubMed, and Google Scholar) and preprint servers (e.g., PsyArXiv and medRxiv) in March 2023, with an update in May 2025.

The following search terms were used: (‘eco’ OR ‘climate’ OR ‘climate change’ OR ‘environment’) AND (‘anxiety’) AND (‘assessment’ OR ‘measurement’ OR ‘measure’ OR ‘scale’ OR ‘tool’ OR ‘inventory’ OR ‘questionnaire’ OR ‘survey’ OR ‘test’). As many papers develop their own measures to address broader research questions, we also conducted a search using the following search terms: (‘eco’ OR ‘climate’ OR ‘climate change’) AND (‘anxiety’).

The *Journal of Climate Change and Health* was hand-searched for relevant articles as it was not available on databases. Reference lists of included articles were manually searched to identify additional articles not captured during electronic database searches. Searches were conducted from Melbourne, Australia.

### 2.2 Eligibility criteria

Searches were limited to full length articles. Restrictions were set to include articles written/translated into English and published from 2006 onwards as eco-anxiety studies were not found before this year [25]. Studies that developed items to examine eco-anxiety or climate change anxiety were eligible, even if the paper did not focus on scale development. If papers assessed multiple eco-emotions, scales or subscales specific to eco-anxiety or climate change anxiety were evaluated. Papers that validated an existing scale in a new population were included. Restrictions were not imposed on sample characteristics, to ensure representation of age ranges and countries.

Non-empirical papers such as reviews, case studies, editorials, and opinion pieces were excluded. Papers that used existing eco-anxiety scales to answer research questions but did not provide additional information about the scales to be reviewed (e.g., psychometric properties) were not included. Papers that assessed related constructs such as climate distress and solastalgia were excluded, as they are conceptualised as distinct from eco-anxiety and climate change anxiety, and therefore outside the scope of the present study [10,26].

### 2.3 Data extraction

Data were extracted from eligible studies regarding article characteristics, sample characteristics, construct measured (eco-anxiety or climate change anxiety), content of items, subscales, development process, and psychometric properties. The first author extracted the data, which was verified by the senior author for accuracy.

### 2.4 Quality assessment/risk of bias

Methodological quality of studies was assessed using a subset of the COSMIN Risk of Bias checklist, as suggested by the developers [24]. This included evaluating whether scale items assessed the intended construct [27], had adequate reliability and validity [28,29] and demonstrated sensitivity and specificity [30,31]. Moreover, cultural influences on the assessment of eco-anxiety were evaluated [32].

Per the COSMIN Risk of Bias checklist, content validity was evaluated based on each scale’s development process [24]. Internal structure was evaluated based on structural validity, internal consistency, and cross-cultural validity/measurement invariance [24]. Each standard was rated as “very good”, “adequate”, “doubtful” or “inadequate” based on quality of evidence. Ratings for each measurement property were determined by the lowest rating of any standard [24].

Next, the Grading of Recommendations Assessment, Development, and Evaluation (GRADE) principles were used [24]. Studies that used or validated the same scale were pooled, and an overall grade was applied. Each scale was graded based on the quality of evidence using one of four labels: “high”, “moderate”, “low”, or “very low”. Labels were based on the number and quality of studies used to validate each scale. For example, a label of “low” would be given to a scale where there is only one study of inadequate quality available, as this is indicative of a serious risk of bias [24].

### 2.5 Data synthesis

Results were synthesised using a narrative synthesis approach [33].

## 3 Results

### 3.1 Search outcomes

**Fig. 1:**
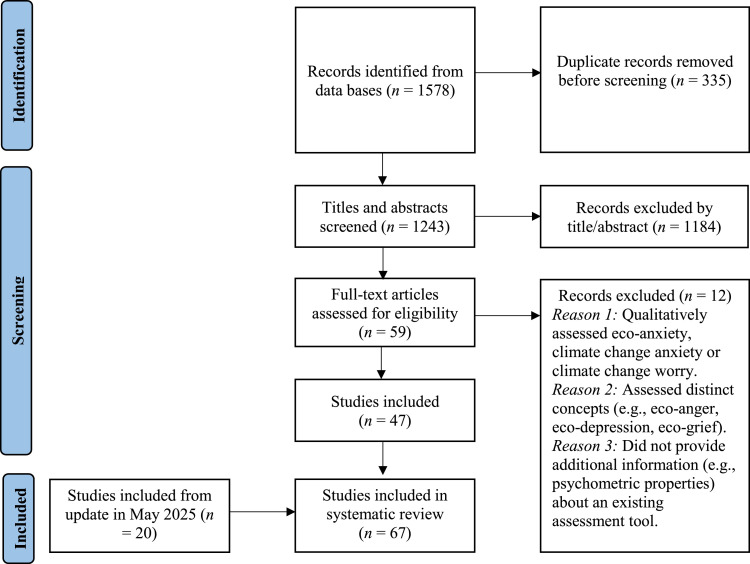
PRISMA flow diagram of the review process.

The database search identified 1578 articles. Of these, 335 were removed as duplicates. Of the remaining 1243 articles, 1184 were deemed irrelevant following title/abstract review. Of the 59 remaining articles, 47 met inclusion criteria based on full-text screening. Reference lists were manually searched to identify papers that were missed during the database search; however, no papers were identified. Twenty articles were identified during an update in May 2025, bringing the total to 67 articles (see Fig. 1). The first, second and senior authors reviewed articles independently and reached consensus about the inclusion of the final 67 articles.

Twelve articles developed a multi-item eco-anxiety scale (*n* = 4) or climate change anxiety scale (*n* t= 8), and 55 articles validated these scales in different populations. Details of each eco-anxiety and climate change anxiety paper are reported in Tables 1 and 2 of the supplementary materials, respectively. The information presented about each scale has been published in the literature. More information about the reviewed scales may be available but were not included in their associated publications.

### 3.2 Quality of reviewed scales

The quality of the 12 scales was evaluated using the COSMIN checklist [24]. For scales used in validation papers, studies were pooled, and a grade was given based on the pooled result. Of these scales, three were deemed to be of "high" quality (25 %): the Hogg Eco-Anxiety Scale [16], the Eco-Anxiety Measurement Scale [34], and the Inventory of Climate Emotions (climate anxiety subscale) [35]. Eight scales were of "moderate" quality (67 %): the Eco-Anxiety Questionnaire [8], the Climate Change Anxiety Scale [9], the Unnamed Climate Anxiety Measure [17], the Climate Emotions Scale [36], the Negative Climate-Related Emotion Scale [37], the Climate Change Distress Scale (climate change anxiety subscale) [26], the Unnamed Adolescent Climate Anxiety Scale [38], and the Hogg Climate Anxiety Scale [39]. One scale (8 %), the Unnamed Eco-Emotions Measure (eco-anxiety subscale) [11] was of “low” quality. The rationale for each quality assessment grade is articulated in Table 3 of the supplementary materials.

### 3.3 Content captured by scales

The items of seven scales (58 %) asked respondents about feelings specific to eco-anxiety, such as worry, anxiety, and fear: the Climate Change Anxiety Scale [9], the Unnamed Eco-Emotions Measure (eco-anxiety subscale) [11], the Hogg Eco-Anxiety Scale [16], the Inventory of Climate Emotions (climate anxiety subscale) [35], the Negative Climate-Related Emotion Scale [37], the Unnamed Adolescent Climate Anxiety Scale [38], and the Hogg Climate Anxiety Scale [39]. The remaining five scales (42 %) included items that captured other eco-emotions such as anger, guilt, depression and hopelessness, in addition to eco-anxiety, despite purporting to measure eco-anxiety or climate change anxiety: the Eco-Anxiety Questionnaire [8], the Unnamed Climate Anxiety Measure [17], the Eco-Anxiety Measurement Scale [34], the Climate Emotions Scale [36], and the Climate Change Distress Scale (climate change anxiety subscale) [26].

Seven scales (58 %) captured behavioural, cognitive and functional dimensions of eco-anxiety, in addition to the affective dimension: the Eco-Anxiety Questionnaire [8], the Climate Change Anxiety Scale [9], the Hogg Eco-Anxiety Scale [16], the Unnamed Climate Anxiety Measure [17], the Eco-Anxiety Measurement Scale [34], the Climate Emotions Scale [36], and the Hogg Climate Anxiety Scale [39]. Five scales (42 %) only captured the affective component of eco-anxiety or climate change anxiety: the Unnamed Eco-Emotions Measure (eco-anxiety subscale) [11], the Inventory of Climate Emotions (climate anxiety subscale) [36], the Negative Climate-Related Emotion Scale [37], the Climate Change Distress Scale (climate change anxiety subscale) [26], and the Unnamed Adolescent Climate Anxiety Scale [38].

### 3.4 Scale development processes

Nine scale development papers (75 %) described in-depth, iterative development processes: the Eco-Anxiety Questionnaire [8], the Climate Change Anxiety Scale [9], the Hogg Eco-Anxiety Scale [16], the Unnamed Climate Anxiety Measure [17], the Eco-Anxiety Measurement Scale [34], the Inventory of Climate Emotions (climate anxiety subscale) [35], the Climate Emotions Scale [36], the Unnamed Adolescent Climate Anxiety Scale [38], and the Hogg Climate Anxiety Scale [39]. The remaining three scales (25 %) which were not written for the purpose of scale development provided no or minimal information about their development process: the Unnamed Eco-Emotions Measure (eco-anxiety subscale) [11], the Negative Climate-Related Emotion Scale [37], and the Climate Change Distress Scale (climate change anxiety subscale) [26].

### 3.5 Psychometric properties

Psychometric properties were rigorously assessed for seven scales (58 %): the Eco-Anxiety Questionnaire [8], the Climate Change Anxiety Scale [9], the Hogg Eco-Anxiety Scale [16], the Eco-Anxiety Measurement Scale [34], the Inventory of Climate Emotions (climate anxiety subscale) [35], Climate Change Distress Scale (climate change anxiety subscale) [26], and the Hogg Climate Anxiety Scale [39]. These papers reported exploratory and/or confirmatory factor analysis and internal consistency. Five of these papers (42 %) additionally assessed test-retest reliability, concurrent validity, and/or discriminant validity [9,16,34,35,39]. Three papers (25 %) only reported internal consistency: the Climate Emotions Scale [36], the Negative Climate-Related Emotion Scale [37], and the Unnamed Adolescent Climate Anxiety Scale [38]. Two papers (17 %) did not report any psychometric properties: the Unnamed Eco-Emotions Measure (eco-anxiety subscale) [11] and the Unnamed Climate Anxiety Measure [17].

The psychometric properties of the Hogg Eco-Anxiety Scale [16] were replicated in 16 papers [[40], [41], [42], [43], [44], [45], [46], [47], [48], [49], [50], [51], [52],[53], [54], [55]]. Conversely, the psychometric properties, particularly the factor structure, of the Climate Change Anxiety Scale [9] were not entirely replicated by independent researchers [[56], [57], [58], [59], [60], [61], [62]], though were replicated in some subsequent papers [58], [63], [64], [65], [66], [67], [68], [69], [70], [71], [72], [73], [74], [75], [76], [77], [78], [79], [80], [81], [82], [83], [84], [85], [86]. Psychometric properties of the Eco-Anxiety Questionnaire [8], the Inventory of Climate Emotions (climate anxiety subscale) [35], the Negative Climate-Related Emotion Scale [37] and the Climate Change Distress Scale (climate change anxiety subscale) [26] were also replicated in additional samples [[63], [87], [88], [89], [90], [91], [92]].

### 3.6 Demographic characteristics of scale development/validation papers

One scale, the Unnamed Adolescent Climate Anxiety Scale, was developed within an adolescent sample [38]. One scale, the Hogg Eco-Anxiety Scale [16], was developed within a sample of young adults/University students. The remaining scales were developed within adult samples: the Eco-Anxiety Questionnaire [8], the Climate Change Anxiety Scale [9], the Unnamed Eco-Emotions Measure (eco-anxiety subscale) [11], the Eco-Anxiety Measurement Scale [34], the Inventory of Climate Emotions (climate anxiety subscale) [35], the Climate Emotions Scale [36], and the Hogg Climate Anxiety Scale [39].

Most scales were developed in Western or European countries: the Eco-Anxiety Questionnaire [8], the Climate Change Anxiety Scale [9], the Unnamed Eco-Emotions Measure (eco-anxiety subscale) [11], the Hogg Eco-Anxiety Scale [16], the Eco-Anxiety Measurement Scale [34], the Inventory of Climate Emotions (climate anxiety subscale) [35], the Climate Emotions Scale [36], the Unnamed Adolescent Climate Anxiety Scale [38], and the Hogg Climate Anxiety Scale [39]. The Eco-Anxiety Questionnaire [8], the Hogg Eco-Anxiety Scale [16], the Climate Change Anxiety Scale [9], the Inventory of Climate Emotions (climate anxiety subscale) [35], and the Hogg Climate Anxiety Scale [39] were validated in several countries. The Climate Change Anxiety Scale [9] was used in 37 countries, the most of any reviewed scale, though its psychometric properties were not replicated in all countries. Table 4 in the supplementary materials lists the countries in which scales have been validated.

## 4 Discussion

This review is the first to systematically identify and evaluate eco-anxiety and climate change anxiety scales across age groups and geographic regions. Sixty-seven articles were evaluated, revealing 12 eco-anxiety or climate change anxiety scales. This review identified key limitations of the eco-anxiety and climate change anxiety scales, which compromise their utility for longitudinal or cross-population studies, including: insufficient content captured by items, inadequate development processes, minimal reporting of psychometric properties, and poor consideration of participants’ demographic characteristics. As eco-anxiety research accelerates, such limitations must be addressed to ensure that eco-anxiety and climate change anxiety are measured accurately. Accurate scales will ensure that these emerging constructs are precisely defined and may facilitate the evaluation of the prevalence of eco-anxiety, associations with wellbeing, and whether management strategies are required. The following sections will elaborate upon these limitations and provide recommendations to improve future eco-anxiety and climate change anxiety scales.

### 4.1 Content captured by eco-anxiety and climate change anxiety scales

Several scales including the Eco-Anxiety Questionnaire [8], the Unnamed Climate Anxiety Measure [17], the Eco-Anxiety Measurement Scale [34], the Climate Emotions Scale [36], and the Climate Change Distress Scale (climate change anxiety subscale) [26] assert to assess eco-anxiety or climate change anxiety, however, their items appear to capture different eco-emotions. For instance, the Eco-Anxiety Questionnaire [8] includes *‘It makes me angry that many people fail to do even the most basic things to protect the environment’*, which appears to be more relevant to eco-anger rather than eco-anxiety, indicating poor content validity. Although eco-anxiety can be related to emotions like eco-anger, it is conceptualised as a distinct construct with its own affective profile and behaviours [8,11,93]. This conflation of affective responses undermines the specificity of scales and poses a challenge for inferring distinct conclusions about eco-anxiety and climate change anxiety.

Moreover, the Unnamed Eco-Emotions Measure (eco-anxiety subscale) [11], the Inventory of Climate Emotions (climate anxiety subscale) [36], the Negative Climate-Related Emotion Scale [37], the Climate Change Distress Scale (climate change anxiety subscale) [26], and the Unnamed Adolescent Climate Anxiety Scale [38] focus solely on the affective component of eco-anxiety/climate change anxiety, failing to capture the multidimensionality of these constructs. Research has demonstrated that eco-anxiety and climate change anxiety encompass affective (e.g., feeling anxious about the consequences of the environmental crisis), behavioural (e.g., day-to-day impacts such as difficulty concentrating) and cognitive components (e.g., rumination about the environmental crisis) [9,16,20]. However, only seven scales (the Eco-Anxiety Questionnaire [8], the Climate Change Anxiety Scale [9], the Hogg Eco-Anxiety Scale [16], the Unnamed Climate Anxiety Measure [17], the Eco-Anxiety Measurement Scale [34], the Climate Emotions Scale [36], and the Hogg Climate Anxiety Scale [39]) captured the multidimensionality of these constructs. Quantifying the functional impact is critical for assessing the burden of eco-anxiety and climate change anxiety without pathologizing these constructs. This is common practice when assessing psychological constructs to identify adults who may need support, such as the Overall Anxiety Severity and Impairment Scale which quantifies both the severity and functional impact of anxiety [94].

### 4.2 Scale development processes

Most of the reviewed papers demonstrated theoretical and methodological rigour by reporting in-depth scale development processes: the Eco-Anxiety Questionnaire [8], the Climate Change Anxiety Scale [9], the Hogg Eco-Anxiety Scale [16], the Unnamed Climate Anxiety Measure [17], the Eco-Anxiety Measurement Scale [34], the Inventory of Climate Emotions (climate anxiety subscale) [35], the Climate Emotions Scale [36], the Unnamed Adolescent Climate Anxiety Scale [38], and the Hogg Climate Anxiety Scale [39]. Gold-standard scale development should involve generating a large item pool based on existing scales and theory, removing and refining items based on expert feedback and pilot testing, and assessing psychometric properties in a large validation sample [95]. However, we were unable to ascertain whether in-depth scale development processes had occurred for the Unnamed Eco-Emotions Measure (eco-anxiety subscale) [11], the Negative Climate-Related Emotion Scale [37], and the Climate Change Distress Scale (climate change anxiety subscale) [26]. Scale development processes should describe how authors selected items, whether they accurately capture eco-anxiety/climate change anxiety, and whether items can be readily understood by respondents. Without this information there can be ambiguity when interpreting items, introduction of biases (e.g., cultural bias), and a lack of generalizability of the scale, which can reduce consistency and replication across studies [27].

### 4.3 Psychometric properties of eco-anxiety and climate change anxiety scales

Though psychometric properties were generally rigorously assessed and reproduced across studies, there is scope to assess these properties more thoroughly. Per best practice guidelines, scales should at least assess content validity, factor structure, internal consistency, and temporal stability [27,30], which most scales adhered to. The Climate Emotions Scale [36], the Negative Climate-Related Emotion Scale [37], and the Unnamed Adolescent Climate Anxiety Scale [38] only reported internal consistency, making it difficult to ascertain their utility beyond reliability between items [96]. Scale developers should strive for rigorous psychometric assessment to support standardization of eco-anxiety scales. Similarly, many scales were validated in additional studies, supporting the utility of eco-anxiety/climate change anxiety scales across populations [19]. This review identified that the Hogg Eco-Anxiety Scale [16] and the Climate Change Anxiety Scale [9] have been most widely used in additional studies. Despite being widely used, the psychometric properties of the Climate Change Anxiety Scale [9] were not entirely replicated, reducing its reliability, utility and validity. Conversely, the psychometric properties of the Hogg Eco-Anxiety Scale [16] were widely replicated, demonstrating its robustness across populations, highlighting the importance of thorough psychometric assessment.

### 4.4 Demographic characteristics of samples

This review identified that many studies developed and validated scales in samples of similar age groups, specifically, young adults/adults. Standardisation and norms should be considered when selecting scales, whereby the population on which a scale was developed/validated should be similar to the respondent [30]. However, this generalisability is limited as several of the reviewed scales were developed within young people or University students, such as the Hogg Eco-Anxiety Scale [16], the Unnamed Climate Anxiety Measure [17], and the unnamed Adolescent Climate Anxiety Scale [38]. Aside from the lack of representation, these restricted samples may be problematic when assessing eco-anxiety which is thought to be more prevalent in younger age groups such as children [12]. However, scales have not been developed for children under 12 years old, despite research suggesting that pre-adolescent children are particularly vulnerable to eco-anxiety due to an increased awareness and concern for the environment [7,97]. As a result, younger age groups may experience different thoughts and feelings related to eco-anxiety which may not be captured by existing scales.

Similarly, most scales were developed in Western or European countries, failing to consider the influence of differing geographical and sociopolitical contexts (e.g., region-specific extreme weather events or varying government climate policy) on mental health [88,98]. Scales are more accurate when respondents are congruent with the country of development or validation. Thus, scales such as the Hogg Climate Anxiety Scale [39] can be used in some Western or European regions but may not yet be suitable for other regions due to a lack of cross-cultural validation. Table 4 of the supplementary materials outlines the countries in which scales have been developed/validated and demonstrates that scales such as the Hogg Eco-Anxiety Scale [16] may be valid in a broader range of geographic locations. Notably, this review revealed that scales have not yet been developed/validated in countries that are particularly vulnerable to the consequences of the environmental crisis (e.g., island nations [99]). This will be critical as these individuals are susceptible to significant affective responses to the environmental crisis [100].

### 4.5 Recommended scales for assessing eco-anxiety and climate change anxiety

Despite the variable quality of existing scales, our findings identify some reliable and valid measures of eco-anxiety and climate change anxiety. Our recommended scale for assessing eco-anxiety in young adults and adults is the Hogg Eco-Anxiety Scale [16] as it had an iterative development process, low risk of bias, high quality of evidence, strong psychometric properties, and cross-cultural replication across several studies. Although the Climate Change Anxiety Scale [9] is the most widely used scale to measure climate change anxiety, this review identified that its psychometric properties were not replicated in several validation studies. As an alternative, the Inventory of Climate Emotions [35] shows promise given its rigorous development, low risk of bias, strong psychometric properties and cross-cultural validity [88]. However, this scale requires further validation to establish its cross-cultural utility and psychometric properties.

### 4.6 Recommendations for future research

The measurement of eco-anxiety and climate change anxiety has strong foundations. However, several limitations were identified across the reviewed scales that should be addressed to facilitate a more accurate assessment of eco-anxiety and climate change anxiety. As such, we propose the following recommendations:

1. Avoid conflation with other eco-emotions by developing scale items that specifically assess eco-anxiety.
2. Ensure scale items comprehensively capture the multifaceted nature of eco-anxiety, including affective, behavioural, cognitive and functional dimensions.
3. Employ rigorous development processes, following established guidelines, such as those by Boateng [27], with transparent reporting.
4. Prioritise the thorough assessment and reporting of psychometric properties to ensure reliability, validity and utility.
5. Expand the focus of scales to include diverse age ranges such as non-University student samples or children under 12 years old.
6. Develop and validate scales across more diverse geographical, political and social contexts, particularly in regions directly impacted by climate change.

### 4.7 Strengths & limitations of this review

A strength of this study was its conceptualisation of climate change anxiety as a component of eco-anxiety. This approach enabled comprehensive insight into the assessment of the overarching concept of eco-anxiety by evaluating climate change anxiety scales, ensuring that all relevant scales were included. An additional strength was its inclusion of eco-anxiety scales across all available age groups and geographic locations. This inclusivity facilitated recommendations and conclusions that are relevant for scales developed for a diversity of populations. Finally, the COSMIN checklist was stringently followed, allowing for an objective assessment of the quality of eco-anxiety scales.

The present review has several limitations that must be acknowledged. First, it may not always be appropriate or feasible for researchers to develop or validate a scale prior to obtaining pilot data, due to location, time and resource constraints. In these situations, it may be necessary to make stepwise increments in the quality of publications. Thus, we acknowledge that it may not have been possible for the authors of the Unnamed Eco-Emotions Measure (eco-anxiety subscale) [11] and the Unnamed Climate Anxiety Measure [17], to publish in-depth validation papers prior to using these scales. Future research should consider using one of the more robust measures available when undertaking research in this field. If they are unable to, they should document why they did not in their publications. Next, this review only considered peer-reviewed journal articles that were published or translated into English, which may have led to the exclusion of relevant articles published in other languages or other forms of media such as theses, government reports and book chapters. Additionally, despite attempts to capture all eco-anxiety and climate change anxiety scales, some papers may not have been identified due to variability in terminology used in the literature.

## 5 Conclusions

This is the first paper to solely evaluate eco-anxiety and climate change anxiety scales across age groups and geographic locations. The present findings align with previous research demonstrating that scales require refinement [12,[20], [21], [22]]. Notably, the present findings extend previous research by demonstrating that the established limitations of eco-anxiety scales in youth [22] also exist in scales for broader age groups. For example, this review identified that many eco-anxiety scales across the lifespan have not been developed with a sufficient level of rigour, nor have their psychometric properties been thoroughly assessed, aligning with previous findings [22]. Additionally, the present findings reiterate past conclusions, demonstrating that the reviewed scales have been developed/validated in limited demographic samples, reducing their generalisability [22].

This review is the first to recommend the most robust scales for assessing eco-anxiety and climate change anxiety based on their content, development processes, psychometric properties and cross-cultural utility. By identifying the most robust scales, we provide a foundation for future research to more accurately assess eco-anxiety. The review also identified limitations regarding scales’ content, development process, psychometric properties, and sensitivity to diverse samples. The proposed recommendations may support a more uniform approach to quantifying eco-anxiety, and consequently, a more precise understanding of eco-anxiety. A more consolidated approach to assessing eco-anxiety may foster more accurate comparison across studies and a more thorough understanding of the consequences of eco-anxiety, such as its relationship with mental health symptoms, and whether management strategies are required.

## CRediT authorship contribution statement

**Sashka K. Samarawickrama:** Writing – review & editing, Writing – original draft, Visualization, Project administration, Methodology, Investigation, Formal analysis, Data curation, Conceptualization. **Sally Richmond:** Writing – review & editing, Supervision, Resources, Methodology, Investigation, Conceptualization. **Nerelie C. Freeman:** Writing – review & editing, Supervision, Resources. **Hannah Kirk:** Writing – review & editing, Supervision, Resources, Project administration, Methodology, Investigation, Formal analysis, Conceptualization.

## Declaration of competing interest

The authors declare that they have no known competing financial interests or personal relationships that could have appeared to influence the work reported in this paper.

## Appendix Supplementary materials

Image, application 1
