# Supplementary material for: Assessing eco-anxiety across the lifespan: A systematic review of current global scales
Source: J Clim Chang Health. 2025 Nov 7;26:100595. doi: 10.1016/j.joclim.2025.100595 (PMC12851308; doi:10.1016/j.joclim.2025.100595)
Supplement: Supplementary file 1 [file mmc1.docx]

**Table 1.**

*Summary of the Content, Development and Psychometric Properties of Eco-Anxiety Scales*

| Authors, Publication Date | Number of Items | Subscales | Sample Characteristics (Age, Country, Sex) | Scale Development Process | Psychometric Properties | Quality Assessment Grade |
| --- | --- | --- | --- | --- | --- | --- |
| **The Eco-Anxiety Questionnaire (Agoston et al., 2022)**  The scale is provided in Appendix A of the paper: https://www.sciencedirect.com/science/article/pii/S2212096322000481 | | | | | | |
| Agoston et al., 2022 | 22 | Two subscales:   - Habitual ecological worry (related to externally focused concerns). - Negative consequences of eco-anxiety (physical, emotional and behavioral consequences). | Development sample:   - 4608 adults from Hungary. - Aged 18+ years old. Average age of 43.3 years. - 57.6% male. | Initial item pool (177 items) was developed based on eco-anxiety literature and interviews with 17 adults affected by climate change. A psychologist reduced this to 93 items. EFA reduced this to the final 22-item measure. | - EFA and CFA verified the two subscales. - Subscales had excellent internal consistency (α of 0.91 and 0.86, respectively). | High |
| Zeier & Wessa, 2024 | 22 | Replicated the two subscales from the original paper. | Validation sample:   - 871 adults from Germany. - Aged 18+ years old. Average age of 49.3 years. - 43.6% male. | N/A (validation paper) | - CFA verified the two subscales. - Subscales had good to excellent internal consistency (α of 0.93 and 0.86, respectively). | N/A (validation paper) |
| Agoston et al., 2024 | 8 | Utilized the two subscales from the original paper. | Validation sample:   - 1000 adults from Hungary. - Aged 18+ years old. Age range and mean age not reported. - 47% male. | Abbreviated the original scale by selecting the four items with the highest factor loadings from each factor (Item 5, 7, 8, 9, 12, 13, 17, and 22). | - Subscales had good internal consistency (α of 0.86 and 0.80, respectively). | N/A (validation paper) |
| Agoston et al., 2024 | 22 | Utilized the two subscales from the original paper. | Validation sample:   - 4685 adults from Hungary. - Aged 18+ years old. Mean age of 43.3 years. Age range not reported. - 57.1% male. | N/A (validation paper) | - Subscales had good to excellent internal consistency (α of 0.91 and 0.86, respectively). | N/A (validation paper) |
| **The Hogg Eco-Anxiety Scale (Hogg et al., 2021)**  The scale is provided in Appendix A of the paper: https://www.sciencedirect.com/science/article/pii/S0959378021001709 | | | | | | |
| Hogg et al., 2021 | 13 | Four subscales:   - Affective symptoms. - Rumination. - Behavioral symptoms. - Anxiety about one’s negative impact on the environment. | Development sample:   - 334 participants for EFA, 365 for CFA, and 189 for test-retest reliability. - University students from Australia and New Zealand. - 17-65 years old. - 40.4% male. | Initial items were developed based on eco-anxiety literature and the Generalized Anxiety Disorder 7-Scale. Items were reduced to 16 items after the first sample. Principal components analysis reduced this to 13 items. | - EFA and CFA verified four subscales. - Subscales had good to excellent internal consistency (α ranged from 0.88 to 0.92). - Test-retest reliability was poor to moderate over a 12-week period. - Strong concurrent and discriminant validity with climate change beliefs, trust in science, and mental health correlates. | High |
| Çimşir et al., 2024 | 13 | Replicated the four subscales from the original paper. | Validation sample:   - 385 participants from Turkey. - 60 participants for test-retest reliability. - 18-65 years old. - 38.7% male. | N/A (validation paper) | - CFA and structural equation modelling verified four subscales. - Overall scale had good internal consistency (α of 0.87). - Subscales had acceptable to good internal consistency (α ranged from 0.74 to 0.85). - Concurrent validity with a range of mental health correlates. | N/A (validation paper) |
| Uzun et al., 2022 | 13 | Replicated the four subscales from the original paper. | Validation sample:   - 698 participants from Turkey. - Aged 18+ years old. Mean age of 23.07 years. Age range not reported. - 27.7% male. | N/A (validation paper) | - EFA and CFA verified four subscales. - Overall scale had excellent internal consistency (α of 0.91). - Subscales had good internal consistency (α ranged from 0.83 to 0.86). | N/A (validation paper) |
| Turkarslan et al., 2023 | 13 | Replicated the four subscales from the original paper. | Validation sample:   - 605 participants from Turkey. - 83 participants for test-retest reliability. - Aged 18-68 years old. - 30% male. | N/A (validation paper) | - EFA and CFA verified four subscales. - Subscales had good to excellent internal consistency (α ranged from 0.82 to 0.93). - Concurrent validity with constructs like mental health correlates. - Good test-retest reliability after 3-weeks. | N/A (validation paper) |
| Heinzel et al., 2023 | 13 | Replicated the four subscales from the original paper. | Validation sample:   - 486 participants from Germany. - Aged 18-73 years old. Mean age of 29.43 years. - 24.9% male. | N/A (validation paper) | - CFA verified four subscales. - Subscales had acceptable to good internal consistency (α ranged from 0.71 to 0.86). - Weak to moderate concurrent and discriminant validity with mental health correlates. | N/A (validation paper) |
| Orrù et al., 2024 | 13 | The authors did not report the factor structure in this paper. | Validation sample:   - 343 participants from Italy. - Aged 18+ years old. Mean age of 29.91 years. - 26.5% male. | N/A (validation paper) | - Overall scale had good internal consistency (α of 0.897). | N/A (validation paper) |
| Rocchi et al., 2023 | 13 | Replicated the four subscales from the original paper. | Validation sample:   - 335 participants from Italy. - Aged 18-73 years old. - 33.7% male. | N/A (validation paper) | - CFA verified four subscales. - Subscales had acceptable to good internal consistency (α ranged from 0.78 to 0.86). - Convergent validity with correlates like pro-environmental behaviors. | N/A (validation paper) |
| Sharma et al., 2023 | 13 | The authors did not report the factor structure in this paper. | Validation sample 1:   - 316 participants from India. - 49% male. - Aged 18+ years old. Age range and mean age not reported.   Validation sample 2:   - 241participants from Italy. - 54% male. - Aged 18+ years old. Age range and mean age not reported. | N/A (validation paper) | - Overall scale had excellent internal consistency across both samples (α of 0.94). | N/A (validation paper) |
| Mathe et al., 2023 | 13 | Replicated the four subscales from the original paper. | Validation sample:   - 275 participants from France. - Aged 18-80 years old. - 31.6% male. | N/A (validation paper) | - CFA verified four subscales. - Subscales had acceptable to good internal consistency (α ranged from 0.73 to 0.88). - Convergent validity with constructs such as climate change anxiety. | N/A (validation paper) |
| Pavani et al., 2023 | 13 | Four original subscales were not replicated.  One factor (eco-anxiety) best fit the data. | Validation sample:   - 167 participants from France. - Aged 18-76 years old. - 35% male. | N/A (validation paper) | - CFA revealed a one-factor structure. - Correlations with variables such as pro-environmental behaviors. | N/A (validation paper) |
| Sampaio et al., 2023 | 13 | Replicated the four subscales from the original paper. | Validation sample:   - 623 participants from Portugal. - 200 participants for test-retest reliability. - Aged 18-25 years old. - 18.5% male. | N/A (validation paper) | - CFA verified four subscales. - Subscales had good to excellent internal consistency (α ranged from 0.85 to 0.92). - Moderate to good reliability over a 7–10-day test-retest period. | N/A (validation paper) |
| Vecina et al., 2024 | 13 | The authors did not report the factor structure in this paper. | Validation sample:   - 403 participants from Spain. - Aged 18-81 years old. - 35.4% male. | N/A (validation paper) | - Overall scale had excellent internal consistency (α of 0.91). - Convergent validity with willingness for environmental behaviors. | N/A (validation paper) |
| Mathers-Jones & Todd, 2023 | 13 | The authors did not report the factor structure in this paper. | Validation sample:   - 96 participants from Australia. - Aged 18-31 years old. - 29.2% male. | N/A (validation paper) | - Overall scale had excellent internal consistency (α of 0.92). - Convergent validity with mental health correlates. | N/A (validation paper) |
| Lutz et al., 2023 | 5: The authors used a subset of items from the original scale. | The authors did not report the factor structure in this paper. | Validation sample:   - 135 participants from Canada. - Aged 17-50 years old. - 26.6% male. | The authors used the four affective items to measure eco-anxiety, and an additional fifth item, to assess eco-anxiety. | - Overall scale had excellent internal consistency (α of 0.91). - Convergent validity with mental health correlates. | N/A (validation paper) |
| Mat & Yilmaz, 2024 | 13 | Utilized the four subscales from the original paper. | Validation sample:   - 374 nursing students from Turkey. - Aged 18-33 years old. - 65% female. | N/A (validation paper) | - Overall scale had excellent internal consistency (α of 0.91). - Subscales had good internal consistency (α ranged from 0.83 to 0.86). | N/A (validation paper) |
| Rodriguez et al., 2024 | 13 | Replicated the four subscales from the original paper. | Validation sample 1:   - 549 Spanish participants. - Aged 16-57 years old. - 86% female.   Validation sample 2:   - 990 Argentinian participants. - Aged 14-89 years old. - 56.8% female. | N/A (validation paper) | - CFA verified four subscales. - Subscales had good internal consistency (α ranged from 0.69 to 0.81). - Subscales had moderate test-retest reliability. | N/A (validation paper) |
| Er et al., 2024 | 13 | Utilized the four subscales from the original paper. | Validation sample:   - 609 nursing students from Turkey. - Aged 18+ years old. Age range and mean age not reported. - 84% female. | N/A (validation paper) | - Excellent internal consistency overall (α of 0.91). - All subscales had good internal consistency (α ranged from 0.80 to 0.85). | N/A (validation paper) |
| **Eco-Anxiety Measurement Scale (Jalin et al., 2023)**  The scale is provided in Table 1 of the paper: https://osf.io/preprints/osf/wnrjx | | | | | | |
| Jalin et al., 2023 | 22 | Three subscales:   - Anxiety-depressive manifestations. - Relational disturbances - Obsession with ecology. | Development sample:   - 262 participants for pre-test sample, 429 for main sample, 90 for test-retest reliability. - Participants from France. - Aged 19-92 years old. - 36.1% male. | Initial items were developed based on themes identified in the authors’ qualitative research. Items were pre-tested with 262 participants, leaving 45 items. Factor analysis reduced this to 22 items. | - Factor analysis revealed three subscales. - Overall scale had excellent internal consistency (α of 0.92). - Convergent validity with correlates such as climate change anxiety and ecological risk perception. - Good temporal stability after a 40-day test-retest period. | High |
| **Unnamed Eco-Emotions Measure (eco-anxiety subscale) (Stanley et al., 2021)**  Scale is provided in the supplementary materials of the original paper: https://www.sciencedirect.com/science/article/pii/S2667278221000018 | | | | | | |
| Stanley et al., 2021 | 6 items form the scale: 2 items measure eco-anxiety. | Overall scale has 3 subscales; one of which is the eco-anxiety subscale. | Development sample:   - 2453 participants from Australia. - Aged 18-87 years old. - 44.2% male. | The authors used data from a larger survey, but did not describe the item development or selection process. | - Confirmatory factor analysis revealed three subscales, one of which is the eco-anxiety subscale. - Convergent validity with a range of mental health correlates. | Low |

*Note.* EFA = exploratory factor analysis, CFA = confirmatory factor analysis.

**Table 2.**

*Summary of the Content, Development and Psychometric Properties of Climate Change Anxiety Scales*

| Reference | | Number of Items | | Subscales | | Sample Characteristics (Age, Country, Sex) | Scale Development Process | Psychometric Properties | Quality Assessment Grade |  | |
| --- | --- | --- | --- | --- | --- | --- | --- | --- | --- | --- | --- |
| **Climate Change Anxiety Scale (Clayton & Karazsia, 2020)**  The scale is provided in Table 1 of the original paper: https://www.sciencedirect.com/science/article/pii/S0272494419307145 | | | | | | | | | | | |
| Clayton & Karazsia, 2020 | | 13 | | Two subscales:   - Cognitive and emotional impairment. - Functional impairment. | | Development sample 1:   - 197 participants from America. - Aged 18-84 years old. - 59.3% male.   Development sample 2:   - 199 participants from America. - Aged 18-84 years old. - 66.8% male. | Items were developed based on literature reviews, understanding personal responses to climate change, and adapting items from existing measures.  Reduced to 13 items following EFA and CFA. | - EFA and CFA revealed two subscales. - Overall scale had excellent internal consistency in both samples (α of 0.92 and 0.93). - Both subscales had good internal consistency (α above 0.80). - Convergent validity with a range of mental health correlates. | | Moderate |  |
| Innocenti et al., 2021 | | 13 | | Two original subscales were not replicated.  A single factor representing overall climate change anxiety was deemed to be the best fit. | | Validation sample:   - 130 participants from Italy. - Aged 19-76 years old. - 32.6% male. | N/A (validation paper) | - CFA did not support the two-factor structure from the original paper. - EFA revealed a single-factor structure. - Both subscales had acceptable internal consistency (α of 0.78 and 0.73). - Convergent validity with a range of mental health correlates. - Good temporal stability after a three-month test-retest period. | | N/A (validation paper) |  |
| Larionow et al., 2022 | | 13 | | Replicated the two subscales from the original paper.  Three subscales also fit the data:   - Intrusive symptoms. - Reflections on climate anxiety. - Functional impairment. | | Validation sample:   - 603 participants from Poland. - Aged 18-70 years old. - 41% male. | N/A (validation paper) | - EFA and CFA verified the two-factor structure and revealed a new three-factor structure. - Subscales had acceptable to excellent internal consistency (α ranging from 0.77 to 0.92). - Convergent and divergent validity with variables such as depressive symptoms. | | N/A (validation paper) |  |
| Wullenkord et al., 2021 | | 13 | | Two original subscales were not replicated.  Two new subscales fit the data:   - Behavioral symptoms. - Cognitive consequences of climate anxiety. | | Validation sample:   - 1011 participants from Germany. - Aged 18-69 years old. - 48.9% male. | N/A (validation paper) | - CFA did not support the original two-factor structure. - EFA revealed a new two-factor structure. - Overall scale had good internal consistency (α of 0.89). - Correlated with variables such as pro-environmental intentions. | | N/A (validation paper) |  |
| Zeier & Wessa, 2024 | | 13 | | Calculated a total score of climate anxiety, as opposed to the original two-factor structure. | | Validation sample:   - 871 participants from Germany. - Aged 18+ years old. Mean age of 49.3 years. - 43.6% male. | N/A (validation paper) | - Overall scale had good internal consistency (α of 0.86). | | N/A (validation paper) |  |
| Reyes et al., 2023 | | 13 | | Utilized the original two subscales from the original paper. | | Validation sample:   - 433 participants from the Philippines. - Aged 18-26 years old. - 33.5% male. | N/A (validation paper) | - Overall scale had excellent internal consistency (α of 0.90). - Both subscales had good internal consistency (α of 0.86 and 0.83). - Correlated with mental health variables. | | N/A (validation paper) |  |
| Simon et al., 2022 | | 13 | | Replicated the two subscales from the original paper. | | Validation sample:   - 452 adolescents from the Philippines. - Age range and mean age not reported. | N/A (validation paper) | - CFA verified two subscales. - Overall scale had excellent internal consistency (α of 0.92). - Both subscales had good to excellent internal consistency (α of 0.90 and 0.85). - Convergent validity was supported. - Discriminant validity was not supported. | | N/A (validation paper) |  |
| Cruz & High, 2022 | | 13 | | Replicated the two subscales from the original paper. | | Validation sample:   - 513 participants from America. - Age range and mean age not reported. - 38.4% male. | N/A (validation paper) | - CFA supported a two-factor structure. Authors note that the subscales cannot be treated as two separate constructs due to high correlations. - Discriminant validity with depression and anxiety. | | N/A (validation paper) |  |
| Tam et al., 2023 | | 13 | | Replicated the two subscales from the original paper. | | Validation sample:   - 1000 participants from America (48% male). - 1000 from China (51% male). - 1000 from India (51% male). - 1000 from Japan (48% male). - All aged 18+ years old. Age range and mean age not reported | N/A (validation paper) | - CFA verified two subscales in all countries. - Both subscales had good to excellent internal consistency in all four countries (α of 0.88-0.95 and 0.87-0.94, respectively). - Correlated with variables such as climate action. | | N/A (validation paper) |  |
| Bratu et al., 2022 | | 13 | | Utilized the original two subscales from the original paper. | | Validation sample:   - 859 participants from Canada. - Aged 16+ years old. Age range and mean age not reported. - 49%-51.7% male. | N/A (validation paper) | - Overall scale had excellent internal consistency (α of 0.94). - Both subscales had good to excellent internal consistency (α of 0.92 and 0.89). | | N/A (validation paper) |  |
| Jang et al., 2023 | | 13 | | Replicated the two subscales from the original paper. | | Validation sample:   - 459 participants from Korea. - Aged 19-65 years old. - 49% male. | N/A (validation paper) | - EFA and CFA verified two subscales. Some items loaded onto different factors, compared to the original paper. - Overall scale had excellent internal consistency (α of 0.91). - Convergent, discriminant, and criterion validity was established. | | N/A (validation paper) |  |
| Shao & Yu, 2023 | | 13 | | The authors did not report the factor structure in this paper. | | Validation sample:   - 523 participants from China. - Aged 16-34 years old. - 55% male. | N/A (validation paper) | - Overall scale had excellent internal consistency (α of 0.95). - Correlated with variables such as pro-environmental behaviors. | | N/A (validation paper) |  |
| Plohl et al., 2023 | | 13 | | Replicated the two subscales from the original paper. | | Validation sample:   - 442 participants from Slovenia. - Aged 18-24 years old. - 22.2% male. | N/A (validation paper) | - CFA verified two subscales. - Both subscales had good to excellent internal consistency (α of 0.90 and 0.86). - Convergent, discriminant and incremental validity with variables such as climate worry. | | N/A (validation paper) |  |
| Whitmarsh et al., 2022 | | 13 | | Utilized the original two subscales from the original paper. | | Validation sample:   - 1338 participants from the United Kingdom. - Aged 18-85 years old. Mean age of 47.1 years. - 46.3% male. | N/A (validation paper) | - Overall scale had excellent internal consistency (α of 0.93). - Correlated with variables such as younger age and anxiety. | | N/A (validation paper) |  |
| Parmentier et al., 2024 | | 13 | | Replicated the two subscales from the original paper. | | Validation sample:   - 431 participants from France. - Aged 18-78 years old. - 27.6% male. | N/A (validation paper) | - Factor analysis verified two subscales. - Both subscales had good internal consistency (α of 0.87 and 0.85). - Correlated with variables such as eco-worry and pro-environmental behaviour commitment. | | N/A (validation paper) |  |
| Nadarajah et al., 2022 | | 13 | | Replicated the two subscales from the original paper. | | Validation sample:   - 369 participants from France. - Aged 18-26 years old. Mean age of 20.05 years. - 73% female. | N/A (validation paper) | - CFA verified two subscales. - Both subscales had adequate internal consistency. - Correlated with variables such as information seeking on climate change. | | N/A (validation paper) |  |
| Mouguiama-Daouda et al., 2022 | | 13 | | Replicated the two subscales from the original paper. | | Validation sample 1:   - 305 participants from France and French-speaking countries. - Aged 17-70 years old. Mean age of 30.8 years. - 72% male.   Validation sample 2:   - 912 participants from France and French-speaking countries. - Aged 17-77 years old. Mean age of 36.9 years. - 44% male. | N/A (validation paper) | - CFA verified two subscales. - Overall scale had good to excellent internal consistency (α of 0.90 in sample 1 and α of 0.87 in sample 2). - Both subscales had acceptable to good internal consistency (α of 0.84 and 0.82 in sample 1, and α of 0.79 and 0.81 in sample 2). - Correlated with constructs such as depression and environmental identity. | | N/A (validation paper) |  |
| Heeren et al., 2022 | | 13 | | Utilized the original two subscales from the original paper. | | Validation sample:   - 2080 participants from France, Belgium, Switzerland, Gabon, Rwanda, Morocco, Algeria, and Congo. - Aged 17-84 years old. - 48.12% male. | N/A (validation paper) | - Overall scale had good internal consistency (α of 0.89). - Both subscales had good internal consistency (α of 0.81 and 0.82). - Correlated with variables such as pro-environmental behaviors. | | N/A (validation paper) |  |
| Feather & Williams, 2022 | | 7 | | The authors did not report the factor structure in this paper. | | Validation sample:   - 771 participants from Australia and New Zealand. - Aged 18-81 years old. - 50.2% male. | Omitted six items from the original scale due to poor face validity. | - The adapted scale had good internal consistency (α of 0.88). - Correlated with variables such as psychological inflexibility, anxiety and depression. | | N/A (validation paper) |  |
| Mejia et al., 2024 | | 4 | | A single factor best fit the items: climate change anxiety. | | Validation sample:   - 1907 participants from Peru, Colombia, Ecuador, Bolivia, Paraguay and Argentina. - Aged 21-29 years old. - 64.5% female. | Eight health professionals evaluated 6 items from the original scale. These items were piloted with 30 participants.  Reduced to four items, following factor analysis. | - CFA revealed a single factor. - Overall scale had good internal consistency (α of 0.88). | | N/A (validation paper) |  |
| Atta et al., 2024 | | 13 | | Replicated the two subscales from the original paper. | | Validation sample:   - 1266 participants from Egypt, Saudi Arabia, Yemen and Palestine. - Age range unspecified. Mean age of 30.9 years. - 62% female. | Utilized the Arabic version of the scale, created by [90] | - Overall scale had adequate internal consistency (α of 0.79). - Both subscales had good internal consistency (α above 0.80). | | N/A (validation paper) |  |
| Fekih-Romdhane et al., 2024 | | 13 | | Replicated the two subscales from the original paper. | | Validation sample:   - 763 participants from Lebanon. - Aged 18+ years old, age range not reported. Mean age of 28.6 years. - 63% female. | Scale was translated into Arabic using the forward-backward method. Items were piloted with 30 Lebanese participants. | - CFA verified two subscales. - Overall scale had excellent internal consistency (α of 0.96). - Correlated with depression, anxiety and stress. | | N/A (validation paper) |  |
| Qin et al., 2024 | | 13 | | The authors examined climate change anxiety as a single variable. | | Validation sample:   - 1851 adolescents from China. - Age range not reported. - 56% female. | N/A (validation paper) | - Overall scale had excellent internal consistency (α of 0.91). - Negatively correlated with pro-environmental behaviours. | | N/A (validation paper) |  |
| Chan et al., 2024 | | 13 | | Replicated the two subscales from the original paper but opted to examine climate change anxiety as a single variable. | | Validation sample 1:   - 1004 adults from America. - 18+ years old. Age range not reported. - 52% female.   Validation sample 2:   - 1009 adults from China. - 18+ years old. Age range not reported. - 49% female. | N/A (validation paper) | - CFA verified a two-factor and one-factor model across both samples. - Correlated with variables such as climate change avoidance, generalized anxiety, depression, self-efficacy, and pro-environmental behaviours. | | N/A (validation paper) |  |
| Garfin & Wong-Parodi, 2024 | | 6 | | Replicated the two subscales from the original paper. | | Validation sample:   - 1479 participants from America. - Age range not reported. Mean age of 51.5 years. - 53% female. | Items were selected based on factor loadings and face validity. | - CFA verified two subscales. - Both subscales had good internal consistency (α of 0.85 and 0.89). | | N/A (validation paper) |  |
| Eren & Yildiz, 2024 | | 13 | | Utilized the two subscales from the original paper. | | Validation sample:   - 419 nursing students from Turkey. - Aged 18-40 years old. - 80% female. | N/A (validation paper) | - Overall scale had acceptable internal consistency (α of 0.78). - Correlated with global climate change awareness. | | N/A (validation paper) |  |
| Cosh et al., 2024 | | 13 | | The authors did not report the factor structure in this paper. | | Validation sample:   - 877 participants from Australia. - Aged 16-25 years old. - 72% female. | N/A (validation paper) | - Overall scale had excellent internal consistency (α of 0.95). | | N/A (validation paper) |  |
| Fekih-Romdhane et al., 2024 | | 13 | | Utilized the two subscales from the original paper. | | Validation sample:   - 596 participants from Lebanon. - Aged 18-35 years old. - 64% female. | N/A (validation paper) | - Overall scale had excellent internal consistency (α of 0.96). | | N/A (validation paper) |  |
| Becht et al., 2024 | | 13 | | Utilized the two subscales from the original paper. | | Validation sample 1:   - 259 adolescents from the Netherlands. - Aged 12-17 years old. - 55% female.   Validation sample 2:   - 267 Colombian and 270 Dutch adolescents. - Aged 12-17 years old. - 51% female. | N/A (validation paper) | - Overall scale had excellent internal consistency across both samples (α of 0.91 and 0.93). - Correlated with pro-environmental behaviours. | | N/A (validation paper) |  |
| Cameron & Kagee, 2025 | | 13 | | The authors did not report the factor structure in this paper. | | Validation sample:   - 343 university students from South Africa. - Age range not reported. 93% were 18-23 years old. | N/A (validation paper) | - Overall scale had excellent internal consistency (α of 0.91). - Correlated with variables such as general anxiety, depression, and climate change concern. | | N/A (validation paper) |  |
| Roberts et al., 2025 | | 6 | | Replicated the two subscales from the original paper. | | Validation sample:   - 1087 participants from the United Kingdom. - Aged 18+ years old, age range not reported. Mean age of 49.5 years. - 46.9% male. | 3 items from each of the two original subscales were selected, following advice provided by the first author of the original paper. | - CFA verified two subscales. - Overall scale had excellent internal consistency (α of 0.96). - Both subscales had excellent internal consistency (α of 0.92 and 0.95). - Correlated with environmentally relevant behavioural intentions. | | N/A (validation paper) |  |
| Prata & Matos, 2025 | | 13 | | Replicated the two subscales from the original paper. | | Validation sample:   - 522 participants from Portugal. - Aged 18-65 years old. - 30% male. | N/A (validation paper) | - EFA and CFA verified two subscales. - Subscales had adequate to excellent internal consistency (α of 0.97 and 0.79). - Correlated with eco-anxiety and mental health correlates. | | N/A (validation paper) |  |
| Pinho et al., 2025 | | 13 | | Utilized the two subscales from the original paper. | | Validation sample:   - 2055 participants from Portugal. - Aged 18-74 years old. - 50.4% male. | N/A (validation paper) | - Overall scale had excellent internal consistency (α of 0.94). - Subscales had good to excellent internal consistency (α of 0.91 and 0.89, respectively). - Correlated with variables such as environmental identity. | | N/A (validation paper) |  |
| **Inventory of Climate Emotions (climate anxiety subscale) (Marczak et al., 2023)**  The scale is provided in the supplementary materials of the paper: https://www.sciencedirect.com/science/article/pii/S0959378023001309#s0130 | | | | | | | | | | |  |
| Marczak et al., 2023 | | 32 items in total. Four items assess climate anxiety | | Eight subscales, with one scale being the climate anxiety subscale. | | Development sample:   - First sample of 632 participants (47.3% male). - Second sample of 300 participants (40.7% male). - All from Poland. - Aged 18-74 years old. Mean age of 39.28 years. | The authors generated a large initial item pool (*n* = 236).  Six experts reduced this to 168 items.  Items were pre-tested with eight participants during interviews. | - EFA and CFA verified eight subscales, one of which is the climate anxiety subscale. - The climate anxiety subscale had good internal consistency (α of 0.86). - Discriminant, concurrent and predictive validity with variables such as emotional reactivity and climate change perceptions. | | High |  |
| Marczak et al., 2024 | | 32 items in total. Four items assess climate anxiety. | | Replicated the eight subscales of the original paper, with one subscale being the climate anxiety subscale. | | Validation sample 1:   - 491 participants from Norway. - Age range not reported. Mean age of 46.8 years. - 50% male.   Validation sample 2:   - 485 participants from Ireland. - Age range not reported. Mean age of 42.6 years. - 39% male.   Validation sample 3:   - 300 participants from Poland. - Age range not reported. Mean age of 40.3 years. - 41% male. | N/A (validation paper) | - CFA verified eight subscales, one of which is the climate anxiety subscale. - The climate anxiety subscale had acceptable to good internal consistency (α of 0.85 in the Norwegian sample and α of 0.77 in the Irish sample). | | N/A (validation paper) |  |
| **Climate Change Distress Scale (climate change anxiety subscale) (Searle & Gow, 2010)**  Items and response format are provided in the paper: https://www.emerald.com/insight/content/doi/10.1108/17568691011089891/full/html | | | | | | | | | | |  |
| Searle & Gow, 2010 | | 9 items form the climate change anxiety subscale. | | Two subscales for the overall scale; one of which is the climate change anxiety subscale. | | Development sample:   - 275 participants from Australia. - Aged 18+ years old. Age range and mean age not reported. - 61% female. | Items were developed by adjusting questions from related studies. | - EFA revealed two subscales, one of which is the climate change anxiety subscale. - Climate change anxiety subscale had excellent internal consistency (α of 0.92). - Correlated with variables such as environmental beliefs. | | Moderate |  |
| Kulcar et al., 2022 | | 9 items form the climate change anxiety subscale, named ‘distress’ in this study. | | Replicated the two subscales from the original paper. | | Validation sample:   - 574 participants from Austria. - Aged 18-30 years old. - The majority were female. | N/A (validation paper) | - Factor analysis verified two subscales. Contrary to the original paper, the authors labelled the climate change anxiety subscale, ‘distress’. - Subscale had excellent internal consistency (α of 0.90). | | N/A (validation paper) |  |
| **Unnamed Climate Anxiety Measure (Hickman et al., 2021)**  The scale is provided in the supplementary material of the paper: https://www.thelancet.com/journals/lanplh/article/PIIS2542-5196(21)00278-3/fulltext#supplementary-material | | | | | | | | | | |  |
| Hickman et al., 2021 | | 8 | | None | | Development sample:   - 1000 participants each from Australia, Brazil, Finland, France, India, Nigeria, Philippines, Portugal, England and America. - Aged 16-25 years old. - 51.4% male. | Items were developed with input from 11 international consultants.  Items were piloted with 17 young people and adjusted according to feedback. | - Correlated with variables such as negative thoughts about climate change, and negative beliefs about government response. | | Moderate |  |
| **Negative Climate-Related Emotion Scale (Ogunbode et al., 2021)**  The items and response format are provided in the paper: https://link.springer.com/article/10.1007/s12144-021-01385-4 | | | | | | | | | | |  |
| Ogunbode et al., 2021 | | 7 | | None | | Development sample:   - Main sample of 10,143 participants from 25 countries (33.7% male). - 1015 from Norway (47.7% male). - Aged 18+ years old. Age range not reported. Mean age of 23.4 years. | Items were developed based on the state anxiety component of the State-Trait Anxiety Inventory (Spielberger, 1983). | - Overall measure had adequate to excellent internal consistency across countries (α ranging from 0.72 to 0.92). - Correlated with variables such as insomnia symptoms. | | Moderate |  |
| Maran & Begotti, 2021 | | 7 | | None | | Validation sample:   - 312 participants from Italy. - Aged 18-28 years old. - 25% male. | N/A (validation study) | - Overall scale had good internal consistency (α of 0.89). - Correlated with variables such as self-efficacy and collective efficacy. | | N/A (validation study) |  |
| Shao & Yu, 2023 | | 7 | | None | | Validation sample:   - 326 participants from China. - Age range and mean age not reported. - 48.12% male. | N/A (validation study) | - Overall scale had excellent internal consistency (α of 0.91). - Correlated with variables such as climate change coverage. | | N/A (validation study) |  |
| **Climate Emotions Scale (Galway & Beery, 2022)**  The scale is available from the authors on request. | | | | | | | | | | |  |
| Galway & Beery, 2022 | Total of 11 items, 1 item asks about anxiety. | | 2 subscales:   - Negative emotions. - Positive emotions. | | Development sample:   - 627 participants from Canada - Aged 18+ years old. Age range not reported. Mean age of 57 years. - 41% male. | | Items were developed based on existing measures, and collaboration with a research advisory group.  Items were pilot tested with 19 members of the public. | - The overall Climate Emotions Scale had good internal consistency (α of 0.89). | | Moderate |  |
| **Unnamed Adolescent Climate Anxiety Scale (Crandon et al., 2024)**  Information about the items is given in the paper, though the exact wording is not provided: https://www.tandfonline.com/doi/full/10.1080/00050067.2024.2404987?needAccess=true#d1e325 | | | | | | | | | | | |
| Crandon et al., 2024 | 3 | | None | | Development sample:   - 261 adolescents from Australia. - Aged 12-20 years old. - 49% female. | | Items were developed based on climate anxiety literature. Items were pilot tested with five Australian adolescents to refine wording. | - The items had good internal consistency (α of 0.79). - Correlated with variables such as avoidance and repetitive thinking. | | Moderate |  |
| **Hogg Climate Anxiety Scale (Hogg et al., 2024)**  The scale is provided in Appendix A of the paper: https://link.springer.com/article/10.1007/s10584-024-03726-1 | | | | | | | | | | | |
| Hogg et al., 2024 | 13 | | 4 subscales:   - Affective symptoms. - Behavioural symptoms. - Ruminative thoughts. - Anxiety about one’s personal impact. | | Development sample 1:   - 501 adults from the United Kingdom. - Aged 18 to 77 years old. - 49% male.   Development sample 2:   - 508 adults from the United States. - Aged 18 to 82 years old. - 50% female. | | Instructions and relevant items of the Hogg Eco-Anxiety Scale [16] were adapted, to refer to climate anxiety. | - Items showed excellent model fit of a 4-factor structure, across both samples. - All subscales were internally consistent in both samples (α ranged from 0.70 to 0.90). - Convergent validity with eco-anxiety, eco-depression, and general worry. | | Moderate |  |

*Note.* EFA = exploratory factor analysis, CFA = confirmatory factor analysis.

**Table 3.**

*Rationales for Quality Assessment Grades for Eco-Anxiety and Climate Change Anxiety Scales*

|  | **Measure** | **Quality Assessment Grade** | **Rationale** |
| --- | --- | --- | --- |
|  | Eco-Anxiety Questionnaire  (Agoston et al., 2022) | Moderate | The authors described an iterative, in-depth scale development process involving pilot testing. The validation study was of very good quality and was conducted with a large sample from Hungary (*n* = 4608). Psychometric properties were sufficiently assessed: the scale had excellent internal consistency and EFA revealed two factors. Factor structure and internal consistency was supported in a German sample and two Hungarian samples, providing preliminary evidence for cross-cultural validity. Additional psychometric properties such as temporal stability could have been assessed in the original paper. |
|  | Hogg Eco-Anxiety Scale  (Hogg et al., 2021) | High | The authors described a rigorous scale development process. The validation study was of very good quality, with several adequately sized samples from Australia and New Zealand (*n* = 334, 365, and 189). Psychometric properties were rigorously assessed, demonstrating excellent internal consistency, moderate temporal stability over a 12-week period, and strong concurrent and discriminant validity. EFA and CFA revealed and confirmed two factors. Psychometric properties and factor structure were confirmed in 16 additional studies conducted in 10 countries, demonstrating cross-cultural validity. |
|  | Eco-Anxiety Measurement Scale  (Jalin et al., 2023) | High | The authors described an iterative, in-depth scale development process which involved pilot testing. The validation study was of very good quality, with several adequately sized samples from France (*n* = 262, 429 and 90). Psychometric properties were rigorously assessed, demonstrating excellent internal consistency, convergent validity, and temporal stability over a 40-day period. Factor analysis revealed three subscales. No other studies cite its use, so cross-cultural validity has not yet been assessed. |
|  | Unnamed Eco-Anxiety Subscale  (Stanley et al., 2021) | Low | The authors did not report how the items were developed or selected. Despite having a large sample size (*n* = 2453), the validation study was doubtful quality due to a lack of transparent reporting. CFA revealed three subscales, one of which is the eco-anxiety subscale. Convergent validity was demonstrated with some mental health correlates. Psychometric properties (e.g., internal consistency and test-retest reliability) were not reported. No other studies cite the use of this measure, so cross-cultural validity has not yet been assessed. |
|  | Climate Change Anxiety Scale  (Clayton & Karazsia, 2020) | Moderate | The authors described an adequate item development process, though items were not pilot tested. The validation study was of adequate quality, with two samples of adequate size (*n* = 197 and 199). Psychometric properties were sufficiently assessed, revealing excellent internal consistency and convergent validity with mental health correlates. EFA and CFA revealed two factors. The scale has been utilized in 37 countries, demonstrating cross-cultural validity. However, the factor structure and psychometric properties were not replicated in all validation studies. Additional psychometric properties such as temporal stability could have been assessed in the original paper. |
|  | Climate Anxiety Subscale from the Inventory of Climate Emotions  (Marczak et al., 2023) | High | The authors described an iterative scale development process involving pilot testing and presenting items to an expert panel. The validation study was of very good quality, with two large samples (*n* = 632 and 300). Psychometric properties were rigorously assessed: EFA and CFA revealed eight subscales, one of which is the climate anxiety subscale. The subscale had good internal consistency, and discriminant, concurrent and predictive validity with several variables. Factor structure and psychometric properties were verified in subsequent Norwegian, Irish and Polish samples, providing preliminary evidence for cross-cultural validity. |
|  | Climate Change Anxiety Subscale  (Searle & Gow, 2010) | Moderate | The authors provided minimal information about the scale development process. The validation study was of adequate quality, with an adequate sample size (*n* = 275). Psychometric properties were sufficiently assessed: EFA revealed two subscales, one of which is the climate change anxiety subscale. The subscale had excellent internal consistency and was correlated with several variables. The psychometric properties were subsequently validated in an Austrian sample, providing preliminary evidence for cross-cultural validity. |
|  | Unnamed Climate Anxiety Measure  (Hickman et al., 2021) | Moderate | The authors described a rigorous development process involving expert input and pilot testing. The validation study was of adequate quality, using a large sample (*n* = 10,000) from 10 countries, demonstrating cross-cultural validity. The authors did not report information about psychometric properties, such as internal consistency, factor structure, and temporal stability. The measure was correlated with several variables. No other studies cite the use of this measure. |
|  | Negative Climate-Related Emotion Scale  (Ogunbode et al., 2021) | Moderate | The authors stated that the items were based on the State-Trait Anxiety Inventory but did not specify how this was done. The validation study was of adequate quality, with a large sample size (*n* = 10,143 and 1015). Minimal information about psychometric properties was reported, demonstrating adequate to excellent internal consistency. The scale was correlated with several variables. The validation study was conducted in 25 countries, demonstrating cross-cultural validity. Subsequent studies have validated the measure in Italy and China, providing further evidence for cross-cultural validity. Additional psychometric properties such as temporal stability and factor analysis could have been assessed in the original paper. |
|  | Climate Emotions Scale  (Galway & Beery, 2022) | Moderate | The authors described a rigorous development process involving input from a research advisory group and pilot testing. The validation study was of adequate quality, with a large sample (*n* = 627). The overall measure had good internal consistency. No other studies cite the use of this measure, so cross-cultural validity has not yet been assessed. |
|  | Unnamed Climate Anxiety Scale for Adolescents (Crandon et al., 2024) | Moderate | The authors described a rigorous development process which included pilot testing. The validation study was of adequate quality, with one sample of adequate size (*n* = 261). Minimal information about psychometric properties were reported, though the items demonstrated good internal consistency. The measure was correlated with several variables. Additional psychometric properties such as temporal stability could have been assessed. No other studies cite the use of this measure, so cross-cultural validity has not yet been assessed. |
|  | Hogg Climate Anxiety Scale (Hogg et al., 2024) | Moderate | The authors provided minimal information about the development process, stating that items from the Hogg Eco-Anxiety Scale (2021) were modified to specifically refer to climate anxiety. The validation study was of high quality, with two samples of large size from the United Kingdom and United States (*n* = 501 and 508), providing preliminary evidence for its cross-cultural validity. Psychometric properties were rigorously assessed, demonstrating acceptable to excellent internal consistency and convergent validity with several variables. Factor analysis revealed four subscales. Additional psychometric properties such as temporal stability could have been assessed. No other studies cite the use of this measure. |

*Note.* EFA = exploratory factor analysis, CFA = confirmatory factor analysis.

**Table 4.**

*Cross Cultural Validation of Eco-Anxiety and Climate Change Anxiety Scales*

| **Name of Scale** | **Countries of Validation** |
| --- | --- |
| Eco-Anxiety Questionnaire [8] | Germany  Hungary |
| Hogg Eco-Anxiety Scale [16] | Argentina  Australia  Canada  France  Germany  India  Italy  New Zealand  Portugal  Spain  Turkey |
| Eco-Anxiety Measurement Scale [33] | France |
| Unnamed Eco-Emotions Measure (eco-anxiety subscale) [11] | Australia |
| Climate Change Anxiety Scale [9] | Algeria  America  Argentina  Australia  Belgium  Bolivia  Canada  China  Colombia  Congo  Ecuador  Egypt  France  Gabon  Germany  India  Italy  Japan  Korea  Lebanon  Morocco  Netherlands  New Zealand  Palestine  Paraguay  Peru  Philippines  Poland  Portugal  Rwanda  Saudi Arabia  Slovenia  South Africa  Switzerland  Turkey  United Kingdom  Yemen |
| Inventory of Climate Emotions (climate anxiety subscale) [34] | Ireland  Norway  Poland |
| Climate Change Distress Scale (climate change anxiety subscale) [37] | Australia  Austria |
| Unnamed Climate Anxiety Measure [17] | America  Australia  Brazil  England  Finland  France  India  Nigeria  Philippines  Portugal |
| Negative Climate-Related Emotion Scale [36] | Australia  Brazil  Canada  Chile  China  Finland  Germany  Italy  Indonesia  Iran  Japan  Malaysia  Netherlands  Nigeria  Norway  Pakistan  Philippines  Portugal  Romania  Russia  Slovakia  Spain  Tanzania  Uganda  United Kingdom |
| Climate Emotions Scale [35] | Canada |
| Unnamed Adolescent Climate Anxiety Scale [38] | Australia |
| Hogg Climate Anxiety Scale [39] | United Kingdom  United States |
